# Atypical *Listeria innocua* strains possess an intact LIPI-3

**DOI:** 10.1186/1471-2180-14-58

**Published:** 2014-03-08

**Authors:** Evelyn M Clayton, Karen M Daly, Caitriona M Guinane, Colin Hill, Paul D Cotter, Paul R Ross

**Affiliations:** 1 Department of Microbiology, University College Cork, Cork, Ireland; 2 Alimentary Pharmabiotic Centre, Cork, Ireland; 3 Teagasc, Moorepark Food Research Centre, Fermoy, Co, Cork, Ireland

## Abstract

**Background:**

*Listeria monocytogenes* is a food-borne pathogen which is the causative agent of listeriosis and can be divided into three evolutionary lineages I, II and III. While all strains possess the well established virulence factors associated with the *Listeria* pathogenicity island I (LIPI-1), lineage I strains also possess an additional pathogenicity island designated LIPI-3 which encodes listeriolysin S (LLS), a post-translationally modified cytolytic peptide. Up until now, this pathogenicity island has been identified exclusively in a subset of lineage I isolates of the pathogen *Listeria monocytogenes*.

**Results:**

In total 64 *L. innocua* strains were screened for the presence of LIPI-3. Here we report the identification of an intact LIPI-3 in 11 isolates of *L. innocua* and the remnants of the cluster in several others. Significantly, we can reveal that placing the *L. innocua lls* genes under the control of a constitutive promoter results in a haemolytic phenotype, confirming that the cluster is capable of encoding a functional haemolysin.

**Conclusions:**

Although the presence of the LIPI-3 gene cluster is confined to lineage I isolates of *L. monocytogenes*, a corresponding gene cluster or its remnants have been identified in many *L. innocua* strains.

## Background

*Listeria monocytogenes* is a food-borne pathogen which is the causative agent of listeriosis [1-5]. It has long been known that the characteristic haemolytic phenotype of *L. monocytogenes* is attributable to the activity of listeriolysin O (LLO), encoded by the *hly* gene located within *Listeria* Pathogenicity Island I (LIPI-1) [5]. However, more recently, it has also been revealed that several strains of lineage I *L. monocytogenes* (of four evolutionary lineages, serotype 4b strains within lineage I have been most commonly associated with outbreaks [6]) (also possess an additional pathogenicity island (designated LIPI-3) which encodes a second haemolysin, designated listeriolysin S [7-9]. Listeriolysin S (LLS) is not normally expressed *in vitro*, and *hly* mutants give a non-haemolytic phenotype on blood agar. LLS is one of a growing number of post-translationally modified cytolysins (post-translationally modified haemolytic peptides) that include the *Streptococcus pyogenes*-associated Streptolysin S (SLS) and the *Clostridium botulinum*/*Clostridium sporogenes*-associated Clostridiolysin S and is a member of the broader family of thiazole/oxazole modified microcins (TOMMs) [9]. It has been established that LLS plays a role in the survival of *L. monocytogenes* in PMNs and also contributes to virulence in the murine model [8]. LIPI-3 consists of 8 genes arranged in the following order: *llsAGHXBYDP.* LlsA is the structural peptide; LlsB, Y and D are enzymes proposed to perform the post-translational modifications; LlsGH is an ABC transporter; LlsP is a protease; while LlsX is of unknown function [7,8]. The associated promoter, P_llsA_, which is situated upstream of *llsA*, is not transcribed in standard laboratory media but is induced by oxidative stress. It has been suggested that expression of the LIPI-3 genes may be induced in the phagosome of macrophages [8]. When P_llsA_ is replaced by a constitutive promoter (P_HELP_), a strongly haemolytic/cytolytic phenotype is revealed under laboratory conditions [8]. The inducible nature of LLS and its absence in many *L. monocyctogenes* strains is probably responsible for the fact that this virulence factor has gone undetected until recently.

*Listeria innocua* is an avirulent species within the Genus *Listeria*. It has been proposed that *L. innocua* and *L. monocytogenes* have evolved from a common ancestor and differ predominantly due to the loss of virulence genes by *L. innocua*[10,11]. This is supported by the existence of atypical *L. innocua* isolates which retain LIPI-1 and other virulence factors [12,13]. In a previous investigation we demonstrated that none of 11 *L. innocua* isolates examined (one of which was initially classified as an *L. grayi* isolate) possessed the equivalent of the LIPI-3 [7,8]. In this study we extended our analysis to a larger collection of strains, which has revealed that several strains possess the remnants of a LIPI-3. In fact, 11 strains possess fully intact LIPI-3 which gives rise to a haemolytic phenotype when the genes are constitutively expressed.

## Methods

### Strains and growth conditions

Tables 1, 2, and 3 list the panel of *Listeria* strains used in this study. Strains were obtained from the Food Microbiology Microbial Collection (University College Cork) and the Special *Listeria* Culture Collection (SLCC). All strains were cultured at 37°C for 16 h in Brain Heart Infusion (BHI) broth or agar (Oxoid, Hampshire, UK) unless otherwise stated. Where necessary, the characterisation of strains as *L. innocua* was confirmed biochemically by means of the API listeria kit (BioMérieux, Lyon, France) and 16S ribosomal DNA (rDNA) with CO1 and CO2 primer pairs previously described by Simpson *et al.*[14]. *Escherichia coli* EC101 was used as an intermediate vector host. Antibiotics were incorporated as follows [8]: Erythromycin (Ery) 150 μg/ml *E. coli,* 5 μg/ml *L. innocua*. Chloroamphenicol (Cm) 10 μg/ml *E. coli* and *L. innocua*. Ampicillin (Amp) 100 μg/ml *E. coli*. 5-bromo-4-chloro-3-indolyl-b-D-galactopyranoside (X-Gal) was incorporated at a concentration of 40 μg/ml.

**Table 1:** **LIPI-3 positive SLCC *L. monocytogenes *** **strains**

**UCC strain ID**	**SLCC strain ID**	Lineage*	**Logged date**	**Source**		**Country of isolation**	**City of isolation**
63	SLCC4352	I	28/04/1975	Human	Spinal fluid	France	Nantes
74	SLCC4563	I	26/11/1975	Human	Unknown	France	Rouen
75	SLCC4330	I	17/03/1975	Human	Spinal fluid	France	Nantes
79	SLCC4309	I	14/02/1975	Human	Liquor	Germany	Munich
86	SLCC3829	I	15/01/1973	Animal	Goat	unknown	Unknown
87	SLCC3734	I	10/11/1972	Food/animal	Milk	Denmark	Copenhagen
89	SLCC4580	I	15/12/1975	Human	Unknown	France	Rouen
94	SLCC3659	I	26/05/1972	Animal	Brain, Sheep	Germany	Frankfurt
101	SLCC6254	I	05/06/1985	Feed	Silage (grass)	Norway	Unknown
102	SLCC6104	I	13/10/1984	Environmental	Sewage	Germany	Unknown
105	SLCC3733	I	10/11/1972	Food/animal	Milk	Denmark	Copenhagen
106	SLCC3606*	I	06/03/1972	Human	Unknown	Belgium	Bruxelles
110	SLCC2503	I	1966	Human	CFS	Germany	Stuttgart
113	SLCC6088	I	13/10/1984	Environmental	Sewage	Germany	Unknown
118	SLCC3834	I	15/01/1973	Animal	Sheep, brain	Germany	Frankfurt
121	SLCC3760	I	24/11/1972	Human	New born, liver	Peru	Lima
133	SLCC6606	I	02/06/1986	Feed	Silage	Switzerland	Unknown
143	SLCC6092	I	13/10/1984	Environmental	Sewage	Germany	Unknown
148	SLCC3732	I	10/11/1972	Food/animal	Milk	Denmark	Copenhagen
154	SLCC3106	I	09/02/1970	Human	Liquor	Germany	Idar-Oberstein
156	SLCC4157	I	09/05/1974	Animal	Cow, Brain	Germany	Freiburg

*Lineages revealed by allele specific oligonucleotide (ASO)-PCR [15].

**Table 2:** ***llsA *** **negative *L. monocytogenes *** **strains**

**UCC strain ID**	**SLCC ID**	Lineage*	**Logged date**	**Source**		**Country of isolation**	**City of isolation**
64	SLCC3996	I	31/08/1973	Human	Spinal fluid	France	Nantes
65	SLCC4410	II	15/07/1975	Human	Blood	France	Nantes
66	SLCC4068	II	08/01/1973	Animal	Red deer, faeces	Germany	Freiburg
67	SLCC6303	II	05/06/1985	Feed	Silage (grass)	Norway	Unknown
68	SLCC6374	II	05/06/1985	Feed	Silage (grass)	Norway	Unknown
69	SLCC6342	II	05/06/1985	Feed	Silage	Norway	Unknown
70	SLCC4274	I	26/11/1974	Human	Unknown	Germany	Freiburg
71	SLCC4280	II	16/12/1974	Unknown	Unknown	Slovak Republic	Bratislava
73	SLCC4063	II	08/01/1974	Animal	Cattle, faeces	Germany	Freiburg
76	SLCC4349	II	28/04/1975	Human	Blood	France	Nantes
77	SLCC4290	II	16/12/1974	Unknown	Unknown	Slovak Republic	Bratislava
78	SLCC4100	II	05/03/1974	Animal	Sheep, brain	Germany	Stuttgart
80	SLCC4481	II	27/10/1975	Unknown	Unknown	Spain	Madrid
81	SLCC4077	II	15/02/1974	Human	Blood	France	Nantes
82	SLCC3852	II	09/04/1973	Animal	Lamb, brain	Germany	Stuttgart
83	SLCC4235	II	16/09/1974	Animal	Hare, caecum	Denmark	Copenhagen
84	SLCC4209	II	12/08/1974	Human	Intestine	Germany	Heidelberg
85	SLCC4230	II	16/09/1974	Animal	Hare, caecum	Denmark	Copenhagen
88	SLCC4592	II	15/12/1975	Human	Unknown	France	Rouen
93	SLCC3738	II	10/11/1972	Animal	Horse	Denmark	Copenhagen
95	SLCC4455	II	10/09/1975	Unknown	Unknown	Hungary	Szolnok
96	SLCC4439	II	10/09/1975	Unknown	Unknown	Hungary	Szolnok
97	SLCC4315	I	14/02/1975	Human	Liquor	Australia	North Adelaide
98	SLCC4234	II	16/09/1974	Animal	Hare, caecum	Denmark	Copenhagen
99	SLCC6108	I	13/10/1984	Environmental	Sewage	Germany	Unknown
100	SLCC643	II	01/01/1958	Human	csf	USA	Georgia
103	SLCC6340	II	05/06/1985	Feed	Silage	Norway	Unknown
104	SLCC293	III	01/01/1955	Unknown	Unknown	USA	Maryland
107	SLCC3631	II	12/04/1972	Animal	Sheep, brain	Germany	Frankfurt
108	SLCC2671	III	01/01/1967	Unknown	Unknown	USA	California
109	SLCC2634	III	1934	Animal	Ruminant	USA	Unknown
111	SLCC6255	II	05/06/1985	Feed	Silage (grass)	Norway	Unknown
112	SLCC6202	II	05/06/1985	Feed	Silage (grass)	Norway	Unknown
114	SLCC6605	II	02/06/1986	Feed	Silage (maize)	Switzerland	Unknown
115	SLCC4138	II	23/04/1974	Animal	Lymph node	Togo	Lome
116	SLCC4617	II	28/12/1975	Unknown	Unknown	Switzerland	Basel
117	SLCC4618	II	28/12/1975	Unknown	Unknown	Switzerland	Basel
119	SLCC4101	II	05/03/1974	Animal	Sheep, brain	Germany	Stuttgart
120	SLCC4070	II	08/01/1974	Animal	Cattle, faeces	Germany	Freiburg
123	SLCC3939	II	09/07/1973	Human	Blood	Belgium	Bruxelles
125	SLCC3847	II	09/04/1973	Animal	Fox, brain	Slovenia	Ljubljana
125	SLCC3864	II	09/04/1973	Animal	Calf, organs	Germany	Freiburg
126	SLCC4079	II	15/02/1974	Human	Meconium	France	Nantes
127	SLCC4294	II	16/12/1974	Unknown	Unknown	Slovak Republic	Bratislava
128	SLCC4442	II	10/09/1975	Unknown	Unknown	Hungary	Szolnok
129	SLCC4444	II	10/09/1975	Unknown	Unknown	Hungary	Szolnok
130	SLCC3278	I	03/09/1970	Animal	Duck, liver	Denmark	Copenhagen
131	SLCC3270	II	03/09/1970	Animal	Hare, pus	Denmark	Copenhagen
132	SLCC3258	II	02/09/1970	Unknown	Unknown	Belgium	Bruxelles
135	SLCC5203	II	17/11/1977	Feed	Silage	Netherlands	Unknown
136	SLCC3683	II	22/06/1972	Environmental	Fir needle	Germany	Unknown
137	SLCC6611	II	02/06/1986	Environmental	Soil	Switzerland	Unknown
138	SLCC4153	I	09/05/1974	Animal	Faeces	Germany	Freiburg
139	SLCC3269	II	03/09/1970	Animal	Hare, spleen	Denmark	Copenhagen
141	SLCC3214	II	18/06/1970	Human	Spinal fluid	France	Lyon
144	SLCC6343	II	05/06/1985	Feed	Silage	Unknown	Unknown
146	SLCC3629	I	04/04/1972	Human	New born; intestine, liver	Peru	Lima
147	SLCC3569	II	08/02/1972	Animal	Hen	France	Alfort
149	SLCC3458	I	08/07/1971	Human	Unknown	France	Rouen
150	SLCC3457	II	08/07/1971	Human	Unknown	France	Rouen
152	SLCC3366	I	11/03/1971	Animal	Pig, brain	Germany	Freiburg
153	SLCC3277	II	03/09/1970	Animal	Bird, liver	Denmark	Copenhagen

*Lineages revealed by allele specific oligonucleotide (ASO)-PCR [15].

**Table 3:** ***Listeria innocua*** **strains used in this study**

**UCC strain ID**	**SLCC strain ID**	**Serotype**	**Logged date**	**Source**		**Country of isolation**	**City of isolation**	***llsA*** **PCR**	**LIPI-3 PCR**
1	SLCC7157*	6a	08/12/1986	Animal	Roe	Switzerland	Bern	✓	✘
2	SLCC7199	6b	18/12/1986	Food	Cheese	Germany	Munich	✓	✓
3	SLCC6483	6b	05/03/1986	Food	Cheese	Switzerland	St.Gallen	✘	✘
4	SLCC6109	6a	13/10/1984	Sewage	Sewage	Germany	Braunschweig	✘	✘
5	SLCC6814	4c	07/05/1986	Human	Liquor (meningitis)	UK	London	✓	✓
6	SLCC6270	6b	05/06/1985	Animal	Goat	Norway	Minde	✓	✘
7	SLCC6276	6b	05/06/1985	Animal	Sheep	Norway	Minde	✓	✓
8	SLCC6362	6b	05/06/1985	Animal	Sheep	Norway	Minde	✓	✘
9	SLCC6370*	6b	05/06/1985	Animal	Sheep	Norway	Minde	✓	✘
10	SLCC6382	6b	05/06/1985	Animal	Sheep	Norway	Minde	✓	✘
11	SLCC6285*	6b	05/06/1985	Feed	Silage (grass)	Norway	Minde	✓	✘
12	SLCC6373	6b	05/06/1985	Feed	Silage (grass)	Norway	Minde	✓	✘
13	SLCC6098	6a	13/10/1984	Sewage	Sewage	Germany	Braunschweig	✘	✘
14	SLCC6007	6a	10/08/1984			Brasil	Rio de Janeiro	✘	✘
15	SLCC6099	6a	13/10/1984	Sewage	Sewage	Germany	Braunschweig	✘	✘
16	SLCC6364	6b	05/06/1985	Animal	Sheep	Norway	Minde	✓	✘
17	SLCC6317*	6b	05/06/1985	Animal	Sheep	Norway	Minde	✓	✘
18	SLCC7030	6a	14/11/1986	Food	Cheese	Germany	Munich	✓	✘
19	SLCC6297*	6b	05/06/1985	Feed	Silage (grass)	Norway	Minde	✓	✘
20	SLCC6356	6b	05/06/1985	Food/animal	Milk	Norway	Minde	✓	✘
21	SLCC6235	6b	05/06/1985	Silage (grass)	Silage (grass)	Norway	Minde	✓	✘
22	SLCC6298	6b	05/06/1985	Feed	Silage (grass)	Norway	Minde	✓	✓
23	SLCC6203	6b	05/06/1985	Silage (grass)	Silage (grass)	Norway	Minde	✓	✓
24	SLCC7116	6a	17/11/1986	Food	Cheese	Austria	Innsbruck	✓	✘
25	SLCC6353	6b	05/06/1985	Food/animal	Milk	Norway	Minde	✓	✘
26	SLCC6409	6b	05/06/1985	Feed	Silage (grass)	Norway	Minde	✓	✘
28	SLCC6541	6a	23/04/1986	Food	Cheese	Germany	Munich	✓	✘
29	SLCC6927	6b	22/09/1986			Austria	Vienna	✓	✘
31	SLCC6228	6b	05/06/1985	Silage (grass)	Silage (grass)	Norway	Minde	✓	✘
30	SLCC6749	6b	31/07/1986	Food	Cheese	Germany	Munich	✓	✓
32	SLCC6322	6a	05/06/1985	Feed	Silage (grass)	Norway	Minde	✓	✘
33	SLCC5916	6a	16/03/1984			Switzerland	Lausanne	✓	✘
34	SLCC5326	6a	09/03/1979			USA	Richmond, Virginia	✓	✘
35	SLCC6283	6b	05/06/1985	Feed	Silage (grass)	Norway	Minde	✓	✘
36	SLCC6246	6b	05/06/1985	Feed	Silage (grass)	Norway	Minde	✓	✘
37	SLCC3533	4b	06/12/2010	Environment	Leaves	Germany	Freiburg	✘	✘
38	SLCC6466	6b	30/01/1986	Food	Cheese	Switzerland	St.Gallen	✓	✓
39	SLCC6359	6b	05/06/1985	Animal	Goat	Norway	Minde	✓	✘
40	SLCC6286	6b	05/06/1985	Feed	Silage (grass)	Norway	Minde	✓	✘
41	SLCC6294	6b	05/06/1985	Animal	Sheep	Norway	Minde	✓	✓
42	SLCC6371	6b	05/06/1985	Animal	Sheep	Norway	Minde	✓	✘
43	SLCC6119	6a	10/12/1984	Human		Germany	Goettingen	✘	✘
44	SLCC3947	4f	27/07/1973	Human		Germany	Cologne	✘	✘
45	SLCC6519	6a	23/03/1986	Food	Cheese	Germany	Munich	✘	✘
46	SLCC6408*	6b	05/06/1985	Feed	Silage (grass)	Norway	Minde	✓	✘
47	SLCC6296	6b	05/06/1985	Feed	Silage (grass)	Norway	Minde	✓	✓
48	SLCC5328	6b	09/03/1979			USA	Richmond, Virginia	✘	✘
49	SLCC6279	6b	05/06/1985	Animal	Sheep	Norway	Minde	✓	✓
50	SLCC6318	6b	05/06/1985	Animal	Sheep	Norway	Minde	✘	✘
51	SLCC6542	6a	23/04/1986	Food	Cheese	Germany	Munich	✓	✘
52	SLCC6272	6b	05/06/1985	Animal	Goat	Norway	Minde	✓	✘
53	SLCC3835*	6b	08/02/1973	Human		Germany	Cologne	✓	✘
54	SLCC5998	6b	16/07/1984	Animal	Cattle	Belgium	Bruxelles	✘	✘
55	SLCC6670	6a	02/06/1986	Food	Milk	Switzerland	Bern	✘	✘
56	SLCC6667	6a	02/06/1986	Food	Milk	Switzerland	Bern	✘	✘
57	SLCC5753*	6b	16/11/1982			Slovak Republic	Bratislava	✓	✘
58	SLCC7113	6b	17/11/1986	Food	Cheese	Austria	Vienna	✘	✘
59	SLCC6103	6b	13/10/1984	Sewage	Sewage	Germany	Braunschweig	✘	✘
60	SLCC6543	6a	23/04/1986	Food	Cheese	Germany	Munich	✓	✘
61	SLCC6977*	4c	13/10/1986	Food	Cheese	Germany	Munich	✓	✓
62	SLCC6921	6a	22/09/1986	Food	Milk	Switzerland	Bern	✘	✘
FH2034	N/A	Unknown	2000	Food	Raw mince	Ireland	Cork	✓	✘
FH1836	N/A	Unknown	2000	Food	Spinach cannelloni	Ireland	Cork	✓	✘
FH2051	N/A	Unknown	2000	Food	Chicken nuggets	Ireland	Cork	✓	✘

*Possess *llsA* but not other LIPI-3 associated genes.

### Sequence analysis

A PCR-based strategy, employing the primer pair *llsA*For-*llsA*Rev, was employed to screen for the presence of the LLS structural gene, *llsA*. These and other primers corresponding to regions both within (1113for, 1114rev, 1115 rev, 1118rev, 1120rev) and surrounding (*araC*rev) the LIPI-3 of *L. monocytogenes* F2365 were employed to amplify flanking DNA sequences which were subsequently sequenced (MWG Biotech) (Table 4). Primer Lin1080_F1, which was designed to amplify from the conserved gene, corresponding to *lin1080* in strain CLIP11262, was used to determine the position of LIPI-3 in *L. innocua* strains relative to this locus. Overlapping sequences were assembled and a consensus sequence was determined using the Seqmanager programme (Lasergene 6) and deposited in Genbank (accession numbers KJ394487, KJ394488, KJ394489 and KJ394490). Putative open reading frames (ORFs) were identified and pair-wise alignment of protein sequences was carried out using Needlemann-Wunsch global alignment algorithms accessed via the European Bioinformatics Institute (EBI) web server. Shading of multiple-aligned sequences was carried out using the Boxshade programme (version 3.2) accessed via the European Molecular Biology web server (EMBnet).

**Table 4:** Primers used in this study

**Primer name**	Sequence (5′ to 3′)*
PllsAchgA(LI)	GG**CTGCAG**AATCCGCGTTCTTG
PllsAchgB(LI)	GAGGTTTTAGGGCTTTGCTC
PhelpFsoe(LI)	*GAGCAAAGCCCTAAAACCTC*GAGATCTGCTGG
PhelpRsoe	*GATGATTGTGATTTAATATTCAT*GGGTTTCACTCTC
PllsAchgC	ATGAATATTAAATCACAATCATC
PllsAchgD	TG**GAATTC**CCAGCTCCATTGTCTC
pORI280For	CTCGTTCATTATAACCCTC
pORI280Rev	CGCTTCCTTTCCCCCCAT
Lin1080_F1	CGGTACGGATTGTGAATGAAGTGG
*llsA*For	CGATTTCACAATGTGATAGGATG
*llsA*Rev	GCACATGCACCTCATAAC
1113for	GTTATGAGGTGCATGTGC
1114rev	GTCTGGGATATGTAGTCC
1115 rev	CACTAGCATGATGTTTATAGGGG
1118rev	CATGACAAGCAGTGCCTGTTGATACAGC
1120rev	CGTTCCCCCTCCTTTTTAGAGCAG
*araC*rev	CTCTCCTTTTCATTAGCCTGC
actA1-f	AATAACAACAGTGAACAAAGC
actA1-r	TATCACGTACCCATTTACC
plcB2-f	TTGTGATGAATACTTACAAAC
plcB2-r	TTTGCTACCATGTCTTCC
actA3-f	CGGCGAACCATACAACAT
plcB3-r	TGTGGTAATTTGCTGTCG

*Restriction site in bold and SOE overhang italicised.

### Constitutive expression of the LIPI-3 cluster of *L. innocua* strain FH2051

The *L. innocua* FH2051 *lls* genes were placed under the control of the strong constitutive synthetic promoter P_HELP_ using the pORI-based *repA*-negative plasmid system as previously described by Cotter *et al*., with some modification [8]. Briefly, P_HELP_ DNA was amplified with the primer pair PhelpFsoe(LI)/PhelpRsoe from the plasmid pPL2luxPHelp [16] and fused between two DNA fragments amplified from the regions flanking P_*llsA*_ by splicing by overlap extension (SOE) PCR [17]. The upstream region was amplified with the primer pair PllsAchgA(LI) and PllsAchgB(LI) and the downstream region was amplified with primers PllsAchgC and PllsAchgD. All PCRs were performed using Vent DNA polymerase (NEB, New England Biolabs, MA, USA). The SOE PCR product was cloned into the multiple cloning site (MCS) of pORI280 following *PstI* and *EcoRI* (NEB) digestion and ligation with the Ligafast rapid DNA ligation system (Promega, Madison, USA). The sequence of the cloned product was verified with MCS primers pORI280For/Rev by MWG Biotech, Germany [18]. Pellet-paint (Novagen) precipitated plasmid was subsequently transformed into the intermediate *repA*-positive host *E. coli* EC101. The plasmid was co-transformed into *L. innocua* FH2051 with the highly temperature-sensitive plasmid pVE6007 which supplies RepA in *trans*. Transformed cells appeared as blue colonies following plating on BHI-Ery-Xgal at 30°C. The integration of pORI280 by single crossover homologous recombination was stimulated by picking a single blue colony from the transformation plate and incubating it on BHI-Ery-Xgal at 30°C for 24 h and subcultured twice on BHI-Ery-Xgal at 42°C. A second crossover event, resulting in the introduction of P_HELP_ in place of P_llsA_ and the eventual loss of the pORI280 vector, was screened for following multiple subcultures in the absence of antibiotic selection. The introduction of P_HELP_ upstream of *llsA* in Ery resistant Cm sensitive colonies was confirmed by PCR. A haemolytic phenotype was determined by spotting 10 μL of an overnight culture of this strain onto Columbia blood agar (Oxoid) containing 5% defibrinated horse blood (TCS Biosciences, Buckingham, UK) and 1 mU/ml sphingomyelinase (Sigma) and examining after 24 h.

### Pulsed- field gel electrophoresis

Pulsed-field gel electrophoresis was carried out following the CDC standardized PulseNet protocol for *L. monocytogenes* with *Asc*I and *Apa*I as the restriction endonucleases. The PFGE patterns were analyzed using BioNumerics software [19].

## Results and discussion

### Screening *L. monocytogenes* and *L. innocua* for homologues of *llsA*

To date LIPI-3 has been identified in ~60% (27 of 46) of lineage I *L. monocytogenes* but was absent from all lineage II (n = 23) and lineage III (n = 5) isolates tested [8]. As a consequence of gaining access to the Seeliger collection of *Listeria* isolates [20], we were provided with the opportunity to screen for the presence of LIPI-3 among an additional 83 *L. monocytogenes* isolates including 30 lineage I, 50 lineage II and 3 lineage III strains. The *llsA* gene was not identified in any lineage II or lineage III strain, consistent with our previous observations (Table 1). However, the *llsA* gene was identified in 70% of lineage I *L. monocytogenes* screened (21 of 30) and, on the basis of PCR amplification, in all cases the full complement of LIPI-3 genes was present. All such isolates originated from human, animal (including milk and feed) and sewage sources. When collated with data from previous studies, it is apparent that 63% (48 of 76) of lineage I isolates are LIPI-3 positive and may be capable of LLS production. All LIPI-3 positive isolates belonged to Lineage I as verified by an allele specific oligonucleotide PCR multiplex (actA1-f, actA1-r, plcB2-f, plcB2-r, actA3-f, plcB3-r) based on the *prfA* virulence gene cluster [15], thus verifying previous observations with respect to the distribution of LIPI-3 among different evolutionary lineages of *L. monocytogenes*[7,8].

Access to the Seeliger collection and other strains also facilitated a further investigation of the LIPI-3 status of *L. innocua*. As stated, a previous analysis of 11 strains of *L. innocua* indicated that all lacked genes associated with LIPI-3 [7,8]. However, screening a larger collection of 64 *L. innocua* strains using *llsA* specific primers revealed that 45 strains (70.3%) were *llsA*-positive (Table 3). Further PCR-based analysis of these isolates, employing a variety of primers designed to amplify across and within the LIPI-3 (*llsA*For, *llsA*Rev*,* 1113for, 1114rev, 1115rev, 1118rev, 1120rev, *araC*rev) revealed that 11 of these strains possess a cluster which is comparable in size, gene content and gene organisation to that of the LIPI-3 cluster found in a subset of lineage I *L. monocytogenes* strains. These 11 isolates originated from a number of European countries between 1984 and 2000, and were isolated from varied sources including processed chicken [1], cheese [7], sheep [7], silage [7] and human [1] (Table 3). Further analysis revealed that 25 *L. innocua* isolates possess a truncated LIPI-3 with no PCR product generated for *llsBYDP*. Sequencing the region confirmed that these genes are absent in at least two isolates (SLCC6270 and SLCC6382). With the exception of *llsP*, these genes have previously been found to be essential for LLS production in *L. monocytogenes*[7]. Of the remaining 28 strains, 9 were found to contain *llsA* but attempts to amplify across or within other LIPI-3 associated genes were unsuccessful and another 19 isolates lacked all LIPI-3 genes.

Two *L. innocua* isolates, SLCC6382 and SLCC6270, containing a truncated LIPI-3, were selected for further analysis. Both SLCC6382 and SLCC6270 shared 98% homology with respect to the structural peptide LlsA. The putative LlsG, LlsH and LlsX proteins from both strains shared 96%, 99% and 95% identity with their *L. monocytogenes* counterpart. *llsB*, *llsY*, *llsD* and *llsP* are absent from both isolates, while the AraC-like regulatory protein determinant was present with 98% identity to the *L. monocytogenes* cluster. As in *L. monocytogenes,* the *L. innocua* cluster is located downstream of a putative glutamine hydrolyzing GMP synthase protein (GuaA). However, the island in SLCC6382 and SLCC6270 commences 600 bases immediately downstream of *guaA* and thus is not flanked by glyoxylase encoding genes, thereby contrasting with LIPI-3 in *L. monocytogenes*.

**Figure 1:**
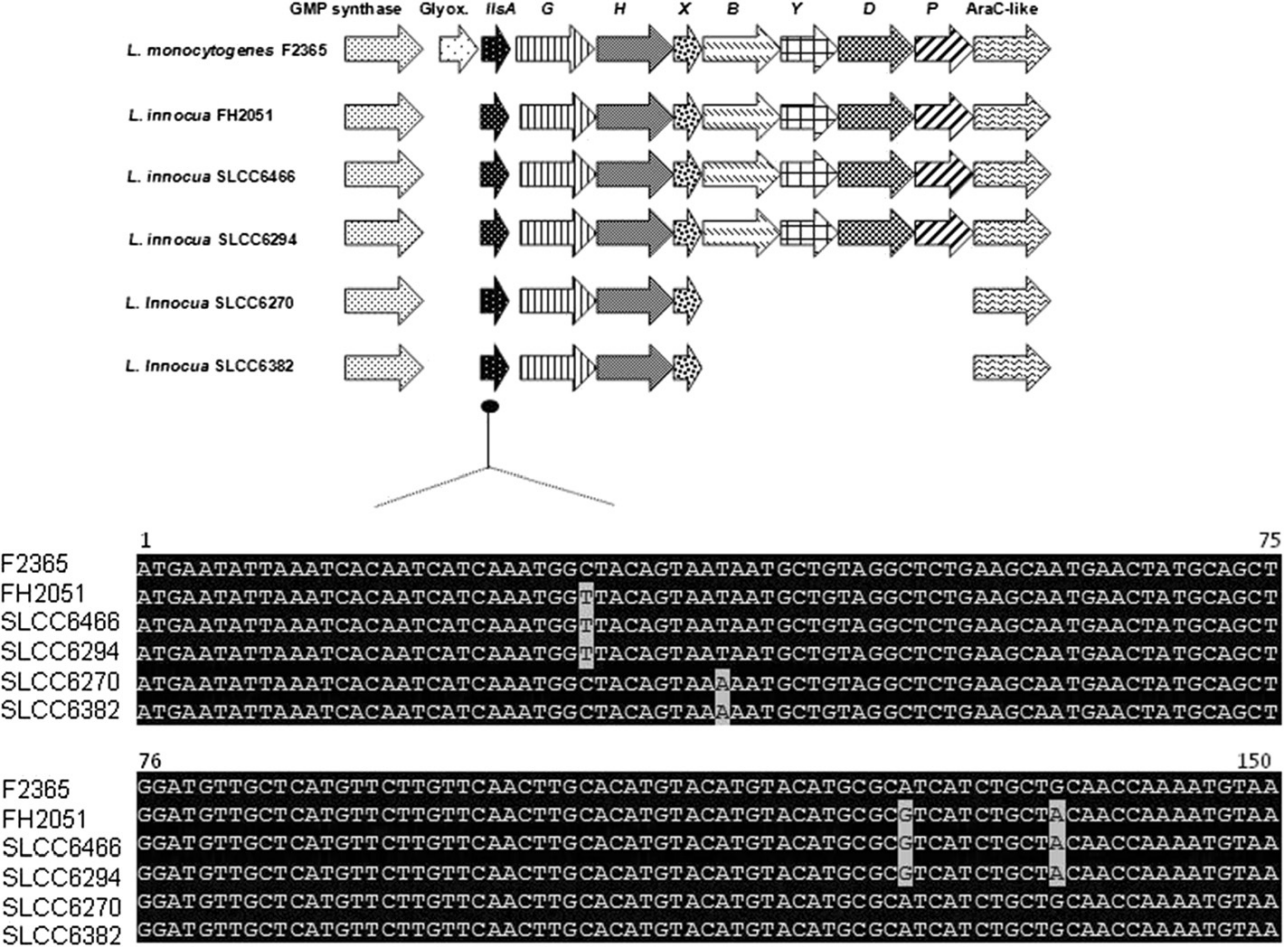
**Alignments of the structural (*llsA*) genes of LIPI-3**_***mono*** **(F2365)**_**and LIPI-3**_***innoc*** **(FH2051, SLCC6466, SLCC6294, SLCC6270 and SLCC6382)**_**.**

**Figure 2:**
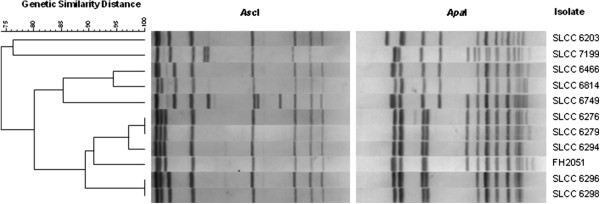
**Dendrograms derived from PFGE profiles of *Asc *I and *Apaf *I macrorestriction displaying restriction pattern similarity among the 11** ***L. innocua *LIPI-3**^**+ **^**isolates.**

Three strains (SLCC6466, SLCC6294, FH2051) possessing an entire LIPI-3 cluster were also selected for a more extensive investigation. Eight complete ORFs were identified, each corresponding to their homologue in the *L. monocytogenes* LIPI-3 cluster (*llsAGHXBYDP*). Sequence alignments confirmed considerable homology at the protein level (Figure 1). The structural peptide LlsA shared 98% homology in the case of the three strains mentioned above to the *L. monocytogenes* equivalent. These *L. innocua* clusters also encode homologs of the putative two component ABC transport system LlsG and LlsH, with LlsG sharing 95.3% (FH2051) and 95% (SLCC6466, SLCC6294) identity, and 98.8% (FH2051) and 99% (SLCC6466, SLCC6294) with respect to LlsH. The putative LlsX homolog, which is of unknown function, is 97% identical to its *L. monocytogenes* counterpart for all three isolates. This gene is believed to be specific to LIPI-3 since no homologue exists among other *sag*-like gene clusters [7]. A corresponding cluster of putative Lls homologs, all of which are predicted to encode biosynthetic enzymes, were also identified [8]; LlsB (99% in the case of all three strains), LlsY (95.4% FH2051, 95% SLCC6466 and SLCC6294) and LlsD (98.4% FH2051, 98% SLCC6466 and SLCC6294). Finally, the *L. innocua* cluster also carries putative LlsP and Lmof2365_1120 homologs, annotated as a CAAX amino-terminal putative metalloprotease and AraC-like regulatory protein which share 93.8% FH2051, 91% SLCC6466 and SLCC6294 and 91.3% FH2051, 94% SLCC6466 and SLCC6294 identity to the *L. monocytogenes* cluster, respectively. PFGE was carried out to assess the relatedness of the 11 *L. innocua* strains harbouring intact LIPI-3 a s. On the basis of this analysis, all LIPI-3^+^ isolates share a high degree of similarity, with the majority of strains (SLCC6466, SLCC6814, SLCC6749, SLCC6276, SLCC6279, SLCC6294, FH2051, SLCC6296 and SLCC6298) displaying 80% similarity and strains SLCC6203 and SLCC7199 sharing 76% identity (Figure 2).

**Figure 3:**
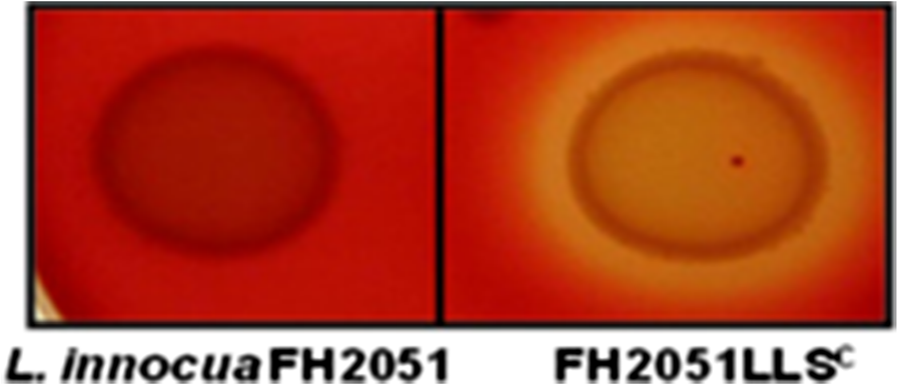
**Growth, after 24 h at 37°C, of *L. innocua *** **FH2051 and FH2051LLS**^**C **^**(10 μL spots of an overnight cultures) on Columbia blood agar containing 5% defibrinated horse blood and 1 mU/ml sphingomyelinase.**

The LIPI-3^+^*L. innocua* FH2051 is non-haemolytic when grown on Columbia blood agar (Figure 1). This is not surprising given that *L. innocua* strains do not produce LLO and the fact that it has previously been established that LLS is not produced by wild type *L. monocytogenes* in laboratory media. It has been established that the latter is due to the fact that P_*llsA*_ is not transcribed under standard laboratory conditions [8]. It has been noted previously that P_*llsA*_ is induced under oxidative stress but, unfortunately, the requirement for an oxidizing agent prevents an assessment of associated haemolytic activity on blood agar [7]. Thus, to investigate the functionality of the LIPI-3 cluster in *L. innocua*, here we constitutively expressed LIPI-3 through the introduction of the constitutive Highly Expressed *Listeria* Promoter [P_HELP_, (LLS^C^)] upstream of *llsA* in *L. innocua* FH2051, to create FH2051LLS^C^. Examination of the resultant strain revealed that the *L. innocua* LIPI-3 is indeed functional as evidenced by a clear haemolytic phenotype on Columbia blood agar (Figure 3).

## Conclusion

In conclusion, we have established that although the presence of the LIPI-3 gene cluster is confined to lineage I isolates of *L. monocytogenes*, a corresponding gene cluster or its remnants can be identified in many *L. innocua*. It is now generally accepted that *L. innocua and L. monocytogenes* evolved from a common ancestor, with *L. innocua* having lost virulence genes since this division. Although rare, *L. innocua* isolates exist which possess the LIPI-1 gene cluster and another *L. monocytogenes* associated virulence gene, *inlA*[12,13]. Nonetheless, the retention of the LIPI-3 cluster by a large proportion of strains is unexpected. The LIPI-3 clusters in the various *L. innocua* strains seem to be at various stages of reductive evolution with a number of stains possessing an intact island, others showing clear evidence of disintegration and yet another group in which the island is completely absent. It is not clear, however, whether the gradual loss of LIPI-3 from *L. innocua* strains is a slow process that has been underway since the existence of the last common ancestor of *L. monocytogenes* and *L. innocua* or if it was initiated following a more recent acquisition of LIPI-3 by *L. innocua* from *L. monocytogenes*.

## Competing interests

The authors have declared that no competing interests exist.

## Authors’ contributions

EC contributed to study design, laboratory investigations, data analysis and manuscript preparation, KD contributed to laboratory investigations, data analysis and manuscript preparation, CG contributed to data analysis, PDC, CH and RPR conceived the study, contributed to study design, data analysis and manuscript preparation. All authors have read and approved the final manuscript.
